# Contextualizing children's rights in sports: the impact of background and contextual factors on Swedish coaches' implementation of the UN convention on the rights of the child

**DOI:** 10.3389/fspor.2026.1820293

**Published:** 2026-05-13

**Authors:** Georgios Pavlogiannis

**Affiliations:** Department of Education, Umeå University, Umeå, Sweden

**Keywords:** children’s sports, coach education, coaching, policy implementation, young athletes

## Abstract

This study draws on Bernstein's theory to examine how background and contextual factors shape coaches' self-reported compliance with the UN Convention on the Rights of the Child (UNCRC). A total of 1,234 coaches completed a questionnaire measuring compliance with five central UNCRC articles and collecting information on coaches' backgrounds and the contexts they work in. Independent samples *t*-tests and one-way ANOVAs were used to examine group differences, and a hierarchical multiple regression analysis was conducted to assess the relative and incremental influence of background and contextual factors on compliance. The group-based analyses showed that both background factors (e.g., coaches' gender, education, and experience) and contextual conditions (e.g., type of sport, type of sports club, and athletes' age and gender) were associated with variation in self-reported compliance. However, the regression analysis demonstrated that contextual factors explained additional variance beyond individual background characteristics, with coaches’ gender, type of sport (team vs. individual), and type of sports club (elite vs. recreational) emerging as the most robust predictors in the final model. Notably, coach education was significant when only background factors were considered, but its effect was no longer significant once contextual factors were included, suggesting that its influence is mediated by coaching environments. The findings underscore the context-specific nature of children's rights implementation in sport and support an understanding of compliance as a pedagogic process shaped by the interaction of formal policy structures (Official Recontextualizing Field) and everyday coaching practices (Pedagogic Recontextualizing Field). The study highlights the importance of attending not only to coach education and policy frameworks, but also to the situated conditions under which children's rights are interpreted and enacted in coaching practice.

## Introduction

Children's rights are firmly established at the policy level worldwide, most notably through the United Nations Convention on the Rights of the Child [UNCRC; (1)]. As the most ratified children's rights convention globally (2), the UNCRC serves for safeguarding children's welfare across various domains, including sports. Previous research underscores the UNCRC's utility in guiding coaches and stakeholders on the appropriate treatment of young athletes, advocating for their active involvement and protection within sports environments (3). However, despite the global recognition and adoption of the UNCRC, its interpretation and implementation can vary significantly depending on the context and the individuals involved in the implementation process. Reynaert et al. (4) argue that children's rights are generally inherently contextual, subject to differing interpretations and realizations among individuals and within diverse settings. Specifically in sports research, Lang (3) further emphasizes the need for research exploring how these rights are perceived and implemented in various sports contexts.

The way policy (e.g., UNCRC) is translated into practice is a complex process involving different levels of interpretation and recontextualization. In this study, Bernstein's (5, 6) concept of the pedagogic device is used as a theoretical lens for understanding this transformation, as it describes how policy knowledge is shaped and communicated through various fields. In sports, coaches play a central role in this process, acting both as recipients of policy and as agents who interpret and implement it based on their own education, experiences, and coaching environments. For example, the level of coach education and the institutional settings (i.e., sports clubs, sports federations) in which they operate—whether within formal coaching programs or informal club structures—affect how the UNCRC is understood and implemented in practice [cf. (6)].

Even in Sweden, the context of this study, there is a wide acceptance and understanding of the UNCRC, but children's rights in sports are still open to interpretation (7). Sweden has employed the UNCRC as a tool to protect children's rights in sports, by ratifying the convention in 1990 (8), including it in their main sport policy document “What Sport Wants” in 2009 (9) and incorporating it in national law in 2020 (10). However, previous research indicates that how coaches follow children's rights in Sweden varies among different sports contexts (7, 11, 12). The policy (e.g., UNCRC) transformation from theoretical knowledge to instructive knowledge to be used in the coaching process takes place both in an official and an informal field. This is what Bernstein (5, 6) calls the Official Recontextualizating Field (ORF) and the Pedagogic Recontextualizating Field (PRF). In Sweden, the ORF is mainly operated by the Swedish Sports Confederation (SSC) and the National Sports Organizations (NSOs), which together are responsible for the official coach education programs. Even if previous research on the content of Swedish coach education is limited, it is expected to align coaching practice with national sport policy goals, including the promotion of children's rights. The PRF, by contrast, operates mainly within sports clubs and everyday coaching environments, where policy principles are further interpreted and implemented through interactions between club representatives, coaches, and athletes. This multilevel structure positions coach education and club practice as key sites for the recontextualization of children's rights in Swedish sport.

Gleaves and Lang (13) argued that there is a lack of knowledge regarding the differences among adults' views related to children's rights across different contexts and coaching backgrounds: coaches of different ages, coaching experience levels, coach education levels and coaches working with athletes of different ages, ability levels, gender, background, or disabilities. From previous research on sports coaching (see *Previous Research*), it is known that there are more contextual and background factors that could possibly influence coaches' compliance with the UNCRC.

This study addresses the lack of research on the different interpretations and implementations of children's rights in sports by investigating how coaches' compliance with the UNCRC is influenced by their background, and the contexts in which they operate. By identifying patterns of variation in compliance across different coaching backgrounds and sporting contexts, the study provides empirically grounded insights that can inform future developments in Swedish coach education by highlighting where policy intentions are recontextualized differently in practice. These insights can support policymakers in reflecting on how children's rights are currently translated through coach education and club environments, and in refining implementation strategies accordingly. Given the near-universal ratification of the UNCRC (2), the question of how children's rights are interpreted and implemented within coaching practice is internationally relevant. While implementation is shaped by national and local conditions, the findings of this study offer analytical insights that may inform research and policy discussions in other national sport systems.

## Previous research

Contextualization or a context-specific approach on children's rights (14) is part of an emerging paradigm within studies of children's rights that has been expressed as, for example, “children's rights from below” (15), “living rights” (16), and “localizing children's rights” (17). This context-specific approach acts as critique towards the existing approach of “children's rights from above” and pays attention to the broader context and conditions of children's rights implementation (14). This current study adopts a context-specific approach on children's rights in sports, challenging the idea that children's rights are applied uniformly by people with different backgrounds and among different contexts (i.e., children's rights from above) and recognizing coaches as active agents with their own understanding and experiences (i.e., children's rights from below) as well as influenced by the local context in which they work (i.e., localizing children's rights).

While this perspective has been explored in broader children's rights studies, its application to sports remains underdeveloped. A recent literature review (18) has showed that previous research on children's rights in sports has primarily focused on risks and violations, such as economic child labour, exploitation, overtraining, and instances of abuse [e.g., (19–22)]. In Sweden, research has highlighted lack of children's rights prioritization within sports clubs [e.g., (7)] and issues like limited athlete participation in decision-making [e.g., (11, 23)] and various forms of abusive behaviours towards young athletes [e.g., (24, 25)].

In contrast, research on coaching has recognized the contextual nature of the coaching process, framing it as a contextual, complex and situation-specific practice influenced by the broader environment (26). Jones et al. (26) considers context acting upon the individuals rather than being in the background of their activities. Moreover, Jones et al. (27) argued that the coaching procedure is mainly a social activity, and coaches are social beings working in a social environment, so their activities must be examined and explained as such. Thus, previous research has conceptualized coaching as a context-specific process.

Empirically, there is previous research showing how coaches' knowledge, beliefs and practices are influenced by background and contextual factors [e.g., (28, 29)] and generally how both individual and contextual factors influence everyday practices in sports clubs [e.g., (30, 31)]. Previous studies have also examined the influence of specific background and contextual factors on coaching, highlighting the impact of the *type of sport* [e.g., (32, 33)], *coach education* [e.g., (28, 34)], *coaches' gender* [e.g., (35, 36)], *coaching experience* [e.g., (37, 38)], *athletes' age* [e.g., (39, 40)], *athletes' gender* [e.g., (33, 41)] and *sports clubs* (28).

Such studies with a context-specific approach directly examining the impact of different background and contextual factors have not fully reached children's rights in sports studies. This encourages the “children's rights from above” perspective, and the idea that the UNCRC impacts coaching in a social vacuum, without considering coaches’ backgrounds, the context they work in and their role as interpreters and translators of the policy.

However, indications of a context-specific approach in children's rights in sports can be detected in previous research, highlighting the need for further investigation. For example, Eliasson (7) found that Swedish coaches and athletes had differing views on the best interests of children in sports, indicating that these rights are subject to individual and contextual interpretations. Similarly, Waerner et al. (12) demonstrated that the structure of equestrian training environments influenced young athletes' ability to participate in decision-making, indicating that children's right to participate in decision-making is influenced by the institutional context (e.g., sports club/stable). Redelius and Eliasson (11) also found that children's involvement in club decisions varied significantly depending on the sport they played. These findings suggest that compliance with the UNCRC is shaped by multiple interacting factors, reinforcing the need for a contextualized approach to children's rights in sports.

Despite these indications, there remains a lack of research examining how coaches' interpretation and implementation of children's rights differ depending on their individual backgrounds and the contexts they work in. Given the complexities of policy recontextualization, further exploration is needed to understand how these elements shape compliance with the UNCRC in different sports settings [cf. (13)].

Thus, this current study aims to be one of the first directly adopting a context-specific approach in children's rights in sports research. Building on prior comparative studies within coaching (e.g., 28–41), this study will compare the level of coaches' compliance with the UNCRC between groups created by the following factors: coaches' gender, education, coaching experience, type of sport, type of sports club as well as athletes' gender and age. These factors represent coaches' individual backgrounds (i.e., background factors: coaches' gender, education, coaching experience) as well as the characteristics of the context they work in (contextual factors: type of sport, type of sports club, athletes' gender and age). This study will therefor provide empirical evidence on how different background and contextual factors influence coaches' compliance with the UNCRC, identifying gaps in compliance and informing sports organizations, policymakers and coach education.

## Aim and research questions

This study aims to investigate coaches' self-reported compliance with the UNCRC, emphasizing the role of background and contextual factors in shaping such compliance. Recognizing coaches as both recipients and agents of policy, this study examines how their individual ideologies shaped by their individual backgrounds, and coaching environments influence their understanding and compliance with the UNCRC.

To achieve this aim, the study addresses the following research questions:

1. How do background (coaches' gender, education, experience) and contextual (type of sport, type of sports club, athletes' age and gender) factors influence coaches' compliance with the UNCRC?
2. How do these factors interact and which exert the strongest influence on coaches' compliance with the UNCRC?

## Theoretical framework

This study examines how children's rights policy (i.e., UNCRC) is interpreted and implemented in different ways depending on background and context. To analyze these variations within the sporting context, Bernstein's concept of the pedagogic device (5, 6) is adopted as a theoretical lens, as it enables an examination of how policy knowledge is transformed as it moves across different institutional and pedagogic fields.

Bernstein (5) describes the policy process as the pedagogic device [cf. (42)]. The pedagogic device describes the rules and procedures followed while policy knowledge is transformed into practical, instructive knowledge (5, 6). The pedagogic device conceptualizes policy not as a fixed entity transmitted unchanged across hierarchical levels, but as knowledge that is produced, recontextualized, and reproduced through a series of structured fields (5, 6). Within the field of production, knowledge is generated and selectively organized into particular textual forms. The field of recontextualization then draws upon this knowledge, transforming it to become instructive. Finally, in the field of reproduction, this recontextualized knowledge is implemented within pedagogic practice.

The UNCRC consists of children's rights knowledge generated in the production field and positioned in international convention, national sports policy and law, before entering sport-specific structures where it is further interpreted and operationalized. This knowledge is subjected to processes of recontextualization, in order to be transformed to sport specific knowledge and then, reproduced via the coaching process. Bernstein (6) argues that there is always a difference between how a text (e.g., UNCRC) was produced in the production field and how it is implemented as a pedagogic practice in the reproduction field. This gap is particularly relevant in sport, where abstract policy principles must be translated into concrete coaching decisions and interactions. One of the reasons for this difference is the fact that the recontextualization of knowledge is ideologically mediated meaning that individual and contextual ideologies shape the way that policy knowledge is being transformed (6).

The data of this study capture the way that coaches' self-reported compliance with the UNCRC is shaped by both their informal coaching environments as well as their individual backgrounds influenced, among others, by the formal structures of Swedish sports operated by the SSC and NSOs. In this respect, Bernstein's (6) distinction between the ORF and the PRF closely corresponds to the formal and informal influences that characterize the sporting context and thus, thereby providing a particularly suitable theoretical and analytical lens for this study. The ORF refers to the state and its agencies—in sports this entails the SSC and NSOs—that select, organize, and reinterpret knowledge. However, the PRF includes informal educational settings—such as sports clubs—where pedagogic meanings are likewise generated and circulated. Within sport, the PRF consists of cultural norms, social interactions, and institutional routines that shape everyday practices in clubs and local sport environments. Accordingly, sports clubs and coaching contexts play a central role in shaping how the UNCRC is interpreted and implemented.

However, according to Bernstein (6), the ORF and the PRF may be guided by distinct and potentially competing ideological positions. In the sporting context, this suggests that coaches may be positioned at the intersection of competing influences, where official policy intentions and localized coaching norms do not always align. This theoretical perspective indicates that variation in coaches' compliance with the UNCRC is not incidental, but a likely outcome of ideologically mediated recontextualization processes within sport.

Taken together, the ORF and PRF provide a theoretically justified framework for analyzing variation in coaches' compliance with the UNCRC within the sporting context examined. Coaches are positioned at the intersection of these two recontextualizing fields, simultaneously influenced by official policy expectations and by localized coaching cultures. This positioning makes variation in implementation not only possible but theoretically expected and analytically meaningful.

According to the theoretical framework guiding this study, variations in UNCRC implementation are anticipated based on individual and contextual factors. However, prior research has not clarified the specific nature of these differences, nor identified which contexts or coaches' characteristics most effectively support UNCRC implementation. Addressing these gaps is a primary objective of the current study, which will also discuss potential explanations for the observed differences.

## Method

### Procedures

The author contacted 20 out of the 30 Swedish NSOs with the largest membership bases (43) aiming to reach NSOs with high participation of children and youth athletes. These NSOs represent central segments of organized children's and youth sport in Sweden. Ten NSOs (see *Participants*) agreed to participate by distributing an online questionnaire to coaches for children and youth. Non-participation was primarily due to organizational capacity and administrative constraints.

In nine cases, NSOs representatives directly distributed the online questionnaire to the coaches. The football NSO, however, provided a secure link for direct email contact. After the initial distribution, two reminder emails were sent, resulting in 1,234 responses from the 8,474 coaches contacted.

The study does not constitute a full-population survey of all Swedish coaches working with children and youth, but rather a large, multi-sport sample drawn from organized, NSO-affiliated coaching contexts. As such, the findings should be interpreted as reflecting patterns within this segment of Swedish sport rather than the entirety of coaching contexts.

### Participants

A sample of 1,234 coaches aged 18–87 (*M* = 42.68, SD = 10.18) participated in this study. More information about the participants is presented in Table 1.

**Table 1:** Description of participants.

Variable	Characteristics	Frequency (*N* = 1,234)	Percent (%)
Coaches’ gender	Male	868	70.5
	Female	363	29.5
Coaches’ years of experience	1–4	425	34.7
	5–9	392	32
	10+	408	33.3
Athletes’ age groups	5–8 years old	103	8.4
	9–12 years old	481	39.1
	13–15 years old	427	34.7
	16–18 years old	218	17.7
Athletes’ gender groups	Boys	438	35.5
	Girls	364	29.5
	Mixed	432	35
Type of sport	Team sports	979	79.3
	Individual sports	255	20.7
Sport	Football	371	30.1
	Basketball	155	12.6
	Handball	128	10.4
	Ice-hockey	125	10.1
	Floorball	93	7.5
	Volleyball	90	7.3
	Swimming	76	6.2
	Skiing	71	5.8
	Tennis	65	5.3
	Wrestling	57	4.6
	Other	3	0.3
Type of sports club	Elite^a^	329	26.8
	Recreational	898	73.2
Coach education	Yes	1,075	87.3
	No	157	12.7
	Level 1^b^	354	35.9
	Level 2	358	36.3
	Level 3	183	18.6
	Level 4	91	9.2

a In individual sport: at least at national championship level—in team sport: at the highest or second highest division at national senior level.

b Level 1 is the lowest level of coach education, usually targeting coaches for young children. The levels increase gradually, with Level 4 being the highest one targeting coaches for elite level.

### Measures

#### Background and context questions

The questionnaire included 20 items covering coaches' background (gender, age, coach education, athletic background, coaching experience, and coaching role) and context (relationship to players, athletes’ age and gender, type of sport and club, number of athletes and coaches, practice frequency, and municipality). The following factors are used analytically; background factors: coaches' gender, coaching experience, coach education, and contextual factors: athletes' gender, athletes’ age, type of sport and type of sports club (i.e., in individual sport: at least at national championship level—in team sport: at the highest or second highest division at national senior level). These were selected based on prior research indicating their potential influence on coaching behaviours (see *Previous Research*).

#### Coaches' compliance with the UNCRC

In the present study, compliance is operationalized as coaches' self-reported frequency of engaging in behaviors that align with selected principles of the UNCRC within their coaching practice. The concept is used in a behavioral and procedural sense, referring to how often coaches report acting in accordance with children's rights-related guidelines, rather than as an indicator of the quality, depth, or ethical embeddedness of those practices.

More specifically, the study draws on a scale measuring coaches' compliance with the UNCRC from a broader questionnaire—developed by the author—assessing coaches' knowledge, awareness, value, and compliance with the UNCRC. The questionnaire's development, validity, and reliability are detailed in a previous publication using the same data set (44). In brief, a confirmatory factor analysis (CFA) was conducted and the model showed satisfactory fit in the first subsample: *χ*^2^_(374)_ = 804.14, *p* < .001, CFI = .91, SRMR = .05, RMSEA = .05, 90% CI [.04, .05], with internal consistency *ω* = .82. Cross-validation in the second subsample confirmed the fit: *χ*^2^_(374)_ = 792.54, *p* < .001, CFI = .91, SRMR = .06, RMSEA = .05, 90% CI [.04, .05], *ω* = .82. Except for the construct validity measured via the CFA reported above, the content validity of the questionnaire was ensured by choosing items used in previous research as well as official national and international guidelines for children's rights in sports [e.g., (45, 46)]. Finally, face validity was ensured through a comprehensive review of the questionnaire conducted both by non-experts and academic professionals, including lecturers, associate professors and professors.

This study uses only the scale measuring coaches' compliance with the UNCRC. While the UNCRC includes 54 articles, four are commonly highlighted as its guiding principles (47): Article 2: All children have the same rights and equal value; Article 3: The best interests of the child shall be taken into account in all decisions concerning children; Article 6: All children have the right to life and development; Article 12: All children have the right to express their opinion and have it respected. Article 19 (Children shall be protected against all forms of physical or mental violence, injury or abuse, neglect or negligent treatment, abuse or exploitation, including sexual abuse) was also included because of its relevance and significance for sports [e.g., (20, 21, 24, 25)]. The questionnaire used in this study includes items measuring coaches' perceived compliance with the five articles mentioned above. The coaches answered on different statements based on a five-point Likert-scale: Never—Rarely—Sometimes—Often—Always (see Table 2).

**Table 2:** Description of the scale about coaches’ compliance with UNCRC.

Scale	Items					
Coaches’ compliance with UNCRC	1	2	3	4	5	It does not apply to me
	Never	Rarely	Sometimes	Often	Always	
Article 2. All children have the same rights and equal value.	I allow certain athletes to have more influence over decision making regarding the training group compared to other athletes.					
	I am fair in my treatment towards all athletes regardless of their background (e.g., gender, ethnicity, religion, etc.).					
	I am patient with all athletes regardless of their performance level.					
Article 3. The best interests of the child shall be taken into account in all decisions concerning children.	I encourage my athletes to participate in other sports.					
	I take the necessary measures against the overtraining effect.					
	I emphasize that what my athletes do in training is much more important than winning.					
	I am considering which is the most appropriate training method for the age of my practitioners.					
Article 6. All children have the right to life and development.	I work with my athletes’ mental development (e.g., stress management, self-confidence, motivation, focus, etc.)					
	I adapt the training to avoid sports-related accidents.					
	I provide children with safe and appropriate means of transport, accommodation and food when they travel to play sports.					
	I inform my athletes about illegal substances such as doping.					
Article 12. All children have the right to express their opinion and have it respected.	I talk to the athletes when I have to solve a sports-related problem.					
	I let the training group set their own goals for the season's competitions (for team sports)/I let each individual athlete set their own goals for the season's competitions (for individual sports).					
	I ask for the athletes’ opinion regarding tactics before a match (for team sports)./I ask for the athletes’ opinion regarding technical and tactical decisions before competition (for individual sports).					
Article 19. Children shall be protected against all forms of physical or mental violence, injury or abuse, neglect or negligent treatment, abuse or exploitation, including sexual abuse.	I follow up on my athletes’ injuries to try to minimize future injuries.					
	I take measures to protect my athletes from all forms of abuse.					
	I use punishment as a method to develop my athletes (e.g., suspension, corporal punishment, fines, etc.).					
	I have witnessed derogatory language among my athletes without reacting.					

Some questionnaire items were negatively worded to capture practices that conflict with children's rights (e.g., favoritism toward certain athletes, failure to respond to derogatory language, or use of punishment). These items were reverse-coded prior to analysis so that higher scores consistently reflected higher levels of self-reported compliance with the UNCRC across all items.

### Statistical analyses

CFA and omega coefficient (*ω*) were calculated with JASP 0.18.3.0, while descriptive statistics, *t*-tests, ANOVAs and the hierarchical multiple regression were conducted via IBM SPSS Statistics 28.0.1.0 software. The level of significance was set at the conventional *p* < .05.

The effect size was calculated through Cohen's *d* in *t*-tests and Eta squared (*η*^2^) in ANOVA, to estimate the magnitude and the practical significance of the reported results (53). The assumption of equal variances was tested using Levene's Test, which showed no significant difference in variances between the groups (see Tables in *Results*).

To examine how background and contextual factors influence coaches' self-reported compliance with the UNCRC as well as whether contextual factors explain additional variance in compliance beyond coaches’ background characteristics, a hierarchical multiple regression analysis was conducted.

In Step 1, background variables were entered into the model, including coach gender, coach education, level of coach education, and coaching experience. In Step 2, contextual variables were added to the model to evaluate their incremental contribution to explaining compliance with the UNCRC. These variables included type of sport (team vs. individual), type of sports club (elite vs. recreational), athletes' age group, and athletes' gender group. The hierarchical structure of the analysis made it possible to examine whether features of the coaching context add explanatory power beyond individual background factors. The dependent variable was coaches' self-reported compliance with the UNCRC.

Prior to analysis, regression assumptions were tested. Visual inspection of residual plots indicated no violations of linearity and homoscedasticity. Residuals were approximately normally distributed, as indicated by the normal probability plot. Multicollinearity was not a concern, with all VIF values below 5. The Durbin–Watson statistic suggested independence of errors. No influential outliers were identified based on standardized residuals and Cook's distance.

## Results

The coaches self-reported that they often comply with the statements constructed for measuring coaches' compliance with UNCRC's articles (*M* = 4.11, SD = .39). Although coaches seem to have a relatively high compliance with the UNCRC, there are differences based on contextual and background factors that can impact coaches' UNCRC compliance. In the following, these findings will be presented in detail.

### Contextuality of coaches' compliance with UNCRC

Independent samples *t*-tests were conducted to compare coaches' compliance with the UNCRC across four binary categories: gender (male–female), coach education (with–without), sport type (team–individual), and club type (elite–recreational). Significant differences were found in all comparisons (*p* < .001; see Table 3). While overall compliance was high, female coaches, those with formal coach education, coaches from individual sports, and those from elite clubs scored significantly higher. These findings suggest that gender, coach education, sport type, and club type may influence compliance. Effect sizes indicated small effects (see Table 3).

**Table 3:** *T*-test results for coaches’ compliance with UNCRC.

Variable	Groups	*M*	Std. deviation	*t*	DF	Sig	*d*	Levene's *F*	Levene's Sig
Coaches’ gender	Male	4.05	.38	7.71	1,229	<.001	.38	.355	.552
	Female	4.24	.38						
Coach education	Yes	4.12	.39	4.09	1,230	<.001	.39	.809	.369
	No	3.99	.42						
Type of sport	Team	4.07	.38	7.28	1,232	<.001	.38	.350	.554
	Individual	4.27	.38						
Type of club	Elite	4.20	.40	4.76	1,225	<.001	.39	.595	.441
	Recreational	4.07	.39						

One-way ANOVAs were conducted to compare coaches' self-reported compliance with the UNCRC across categories with more than two groups. Although overall compliance was high, significant group differences were found (*p* < .001; see Table 4). Tukey *post hoc* tests showed that coaches with 5–9 years of experience scored significantly lower than those with 1–4 or 10+ years of experience. Coaches with the highest level of coach education (i.e., Level 4) scored significantly higher than the other groups. Coaches of mixed-gender athlete groups scored significantly higher than those coaching only boys or only girls. Coaches of the youngest athletes (i.e., 5–8-year-olds) scored significantly higher than those coaching older age groups. These findings suggest that coaching experience, coach education level, and the gender and age of athletes may influence compliance with the UNCRC. Effect sizes (*η*^2^) were small (see Table 4).

**Table 4:** One-way ANOVA results for coaches’ compliance with UNCRC.

Variable	Groups	*M*	Std. deviation	*F*	DF	Sig	*η* ^2^	Levene's *F*	Levene's Sig
Coaching experience	1–4 years	4.12	.39	8.41	21,222	<.001	.01	1.48	.226
	5–9 years	4.04	.40						
	10+ years	4.15	.37						
Coach education	Level 1	4.11	.40	7.37	3,971	<.001	.02	2.00	.111
	Level 2	4.08	.36						
	Level 3	4.12	.37						
	Level 4	4.30	.35						
Athletes’ gender group	Boys	4.03	.40	23.83	21,231	<.001	.03	.796	.451
	Girls	4.08	.36						
	Mixed	4.21	.39						
Athletes’ age group	5–8	4.31	.41	10.98	31,225	<.001	.03	1.52	.207
	9–12	4.08	.40						
	13–15	4.08	.36						
	16–18	4.11	.40						

### Hierarchical regression analysis of background and contextual factors of coaches' self-reported compliance with the UNCRC

A hierarchical multiple regression was conducted to examine how background and contextual factors influence coaches' self-reported compliance with the UNCRC, which factors are the most significant and whether contextual factors explain additional variance in coaches' compliance with the UNCRC beyond background characteristics.

In Step 1, background variables (coaches' gender, coach education and its levels as well as coaching experience) significantly predicted compliance, explaining 5.4% of the variance (*R*^2^ = .054), *F*_(4, 1,156)_ = 13.62, *p* < .001.

In Step 2, contextual variables (type of sport, type of sports club, athletes' age, and athletes' gender) were added, resulting in a significant increase in explained variance (Δ*R*^2^ = .030), Δ*F*_(4, 952)_ = 7.70, *p* < .001. The final model explained 8.4% of the variance in compliance (*R*^2^ = .084), *F*_(8, 952)_ = 10.91, *p* < .001.

This indicates that, while both background and contextual factors influence coaches’ self-reported compliance with the UNCRC, contextual factors contribute significantly to explaining compliance beyond coaches' background characteristics.

In the final model, coach gender (*β* = .192, *t* = 5,897, *p* < .001), type of sport (*β* = .130, *t* = 3.319, *p* < .001), and type of sports club (*β* = .064, *t* = 1.974, *p* = .049) emerged as significant predictors of compliance with the UNCRC.

In contrast, coach education, level of education, coaching experience, athletes' age and athletes' gender were not significant. Notably, level of coach education was significant in the initial model (*β* = .015, *t* = 3.087, *p* = .002) but lost significance in the final model, suggesting that its effect is explained by contextual variables.

Overall, these findings indicate that contextual factors play a more important role than background characteristics in predicting compliance.

### Regression's most significant factors: coaches' gender—type of sport—type of club

Coaches' gender seems to influence to some extend all the examined articles of the UNCRC (see Table 5). More specifically, female coaches reported to prioritize the best interest of the child and children's health in sports, to be more thoughtful regarding the overtraining effect, sports-related accidents, following up young athletes' injuries, adapting the training to the athletes' age and providing safe and appropriate transportation, accommodation and nutrition to children during sports travels. They, also, reported to balance better a children's rights coaching perspective with performance- and win-directed coaching behaviours compared to male coaches, since they score higher on behaving equally to all athletes regardless of their performance level, emphasizing that training is more important than winning and including children in the decision-making process regarding technical and tactical decision before games and competitions.

**Table 5:** Biggest differences on individual items of the questionnaire based on coaches’ gender.

UNCRC Article No	Items	*Μ*_female_ (SD)	*Μ*_male_ (SD)
2	I am patient with all athletes regardless of their performance level.	4.57 (.58)	4.38 (.74)
3	I take the necessary measures against the overtraining effect.	4.59 (1.17)	4.33 (1.18)
3	I emphasize that what my athletes do in training is much more important than winning.	4.25 (.82)	4.00 (.95)
3	I am considering which is the most appropriate training method for the age of my practitioners.	4.63 (.57)	4.48 (.68)
6	I adapt the training to avoid sports-related accidents.	4.37 (.77)	4.11 (.90)
6	I provide children with safe and appropriate means of transport, accommodation and food when they travel to play sports.	4.52 (.97)	4.10 (1.25)
12	I ask for the athletes’ opinion regarding tactics before a match (for team sports)./I ask for the athletes’ opinion regarding technical and tactical decisions before competition (for individual sports).	3.31 (.97)	3.11 (1.00)
19	I follow up on my athletes’ injuries to try to minimize future injuries.	4.18 (.85)	3.99 (.92)
19	I have witnessed derogatory language among my athletes without reacting (Reverse-worded).	4.65 (.72)	4.46 (.81)

SD, standard deviation; *M*_female_, mean scores for female coaches; *M*_male_, mean scores for male coaches.

Type of sports influences coaches' self-reported compliance with the UNCRC in its own way (see Table 6). Individual sports coaches reported to be more aware of the overtraining effect, sports related accidents and doping, while they score higher on including athletes in discussions regarding technical and tactical decision before competitions compared to team sports coaches. Finally, type of sports seems to have an impact on UNCRC's Article 19, since individual sports coaches reported to take measures to protect their athletes from abuse more often, and to use punishment in their trainings less often than team sports coaches.

**Table 6:** Biggest differences on individual items of the questionnaire based on type of sport.

UNCRC Article No	Items	*Μ*_individual_ (SD)	*Μ*_team_ (SD)
2	I allow certain athletes to have more influence over decision making regarding the training group compared to other athletes (Reverse-worded)^a^.	4.12 (.88)	3.96 (.91)
2	I am patient with all athletes regardless of their performance level.	4.61 (.63)	4.39 (.71)
3	I take the necessary measures against the overtraining effect.	3.54 (1.26)	3.35 (1.16)
6	I adapt the training to avoid sports-related accidents.	4.50 (.69)	4.10 (.89)
6	I inform my athletes about illegal substances such as doping.	3.17 (1.46)	2.52 (1.48)
12	I ask for the athletes’ opinion regarding tactics before a match (for team sports)./I ask for the athletes’ opinion regarding technical and tactical decisions before competition (for individual sports).	3.41 (1.04)	3.11 (.98)
19	I take measures to protect my athletes from all forms of abuse	4.52 (.94)	4.29 (1.08)
19	I use punishment as a method to develop my athletes (e.g., suspension, corporal punishment, fines, etc.) (Reverse-worded)^a^	4.82 (.46)	4.67 (.64)

^a^ Reverse-worded items refer to items formulated in the opposite direction of the construct and were reverse-coded before computing scale scores.

SD, standard deviation; *M*_individual_, mean scores for coaches of individual sports; *M*_team_, mean scores for coaches of team sports.

Type of sports club seems to influence coaches' self-reported compliance with the UNCRC (see Table 7), since coaches from elite sports clubs report to protect their young athletes from injuries, sports related accidents as well as doping and other illegal substance more often than coaches from recreational clubs, taking into greater consideration appropriate training methods for their athletes' age and working more often with their athletes' mental development. Notably, coaches from elite clubs report that they more often allow athletes to decide their own goals for the sports season.

**Table 7:** Biggest differences on individual items of the questionnaire based on type of sports club.

UNCRC Article No	Items	*Μ*_elite_ (SD)	*Μ*_recreational_ (SD)
3	I am considering which is the most appropriate training method for the age of my practitioners.	4.49 (.68)	4.32 (.79)
6	I work with my athletes’ mental development (e.g., stress management, self-confidence, motivation, focus, etc.)	3.98 (.90)	3.81 (.90)
6	I adapt the training to avoid sports-related accidents.	4.33 (.83)	4.13 (.88)
6	I inform my athletes about illegal substances such as doping.	3.10 (1.57)	2.49 (1.44)
12	I let the training group set their own goals for the season's competitions (for team sports)/I let each individual athlete set their own goals for the season's competitions (for individual sports).	3.78 (1.15)	3.33 (1.23)
19	I follow up on my athletes’ injuries to try to minimize future injuries.	4.21 (.89)	3.98 (.90)

SD, standard deviation; *M*_elite_, mean scores for coaches from elite clubs; *M*_recreational_, mean scores for coaches from recreational clubs.

## Discussion

The results of this study endorse a context-specific perspective regarding children's rights in sports, aligning with prior research on children's rights across other settings [e.g., (14–17)], earlier investigations into the contextual aspects of coaching [e.g., (26, 27)], as well as Bernstein's (5) pedagogic device, which incorporates individual and contextual factors into policy recontextualization and implementation. Notably, this study advances the discourse by clarifying how background and contextual elements shape coaches self-reported compliance with the UNCRC, identifying the factors that appear most significant, and highlighting which coaching contexts and coaches' background characteristics facilitate or hinder effective UNCRC implementation. The following section will discuss these new findings alongside a discussion of potential explanations.

Taken together, the findings indicate that both individual background and contextual factors are associated with variation in coaches' self-reported compliance with the UNCRC, including coaches' gender, coaching experience, coach education and its level, athletes' age and gender, as well as type of sport and type of sports club. Rather than reiterating these differences descriptively, the *Discussion* focuses on what these patterns reveal about the pedagogic processes through which children's rights are interpreted and implemented in sports. Notably, higher compliance was most consistently associated with female coaches; coaches with extensive experience; those who have completed coach education, particularly at the highest available level; coaches of younger children (ages 5–8); coaches of mixed-gender groups; individual-sport coaches; and coaches working within elite sports clubs.

From the perspective of Bernstein's pedagogic device (5), these findings position coaches not only within the field of reproduction, where policy is implemented in practice, but also within processes of recontextualization. Coaches appear to decode and recode children's rights in relation to their own pedagogic orientations and the expectations embedded within their coaching environments before reproducing these interpretations in everyday practice. This helps explain why strong formal commitments to the UNCRC at national policy level do not automatically result in uniform implementation across sports or coaching contexts. Few previous studies from the research field of children's rights in sports have also indicated some differences, focusing though primarily on the different UNCRC implementations among different sports and different sports clubs (11, 12). Thus, this study extends the discourse on variation in children's rights implementation by considering a broader set of individual background and contextual factors, as well as how these factors relate to one another within coaching practice.

### Individual background factors and the interpretation of the UNCRC

The influence of individual background variables—particularly gender, coach education, and coaching experience—suggests that compliance with the UNCRC is closely tied to coaches' pedagogic dispositions rather than simply their exposure to policy texts. In the present study, higher levels of self-reported compliance were consistently associated with female coaches, coaches with extensive experience, and those with higher levels of coach education.

These patterns suggest that optimal UNCRC implementation is likely to occur in coaching environments where practitioners have both formal pedagogical preparation and substantial practical experience. Higher levels of coach education may provide a stronger grounding in child-centered pedagogies, safeguarding principles, and rights-based coaching approaches, enabling coaches to recognize and respond appropriately to children's needs. Likewise, experienced coaches may be better equipped to translate these principles into everyday interactions, having developed more refined relational skills and situational judgement over time. At a general level, these findings might indicate that both the ORF (i.e., coach education) and PRF (i.e., experience from coaching within sports clubs) contribute to the recontextualization of the UNCRC in a way that supports coaches' compliance with the UNCRC.

However, the hierarchical regression analysis nuances this interpretation by demonstrating that background factors alone explain a limited proportion of variation in compliance, and that several individual characteristics—most notably coach education and coaching experience—lose predictive significance once contextual factors are taken into account. This finding suggests that the influence of individual background characteristics does not operate in isolation, but is conditioned by the contexts in which coaching practice is enacted. From a Bernsteinian perspective (6), this indicates that the effects of the ORF—such as formal coach education—may be mediated through the PRF, rather than directly translated into practice. In other words, exposure to official pedagogic discourse does not automatically generate higher compliance with children's rights unless it is supported, reinforced, or made meaningful within local coaching environments. The loss of significance of coach education once contextual factors are included thus provides empirical support for the argument that recontextualization processes within clubs and sport-specific settings play a decisive role in shaping how policy knowledge is enacted.

However, according to Bernstein (6), ORF and PRF have their own ideological positions, which may contradict each other, fighting for control over the field; in this case the coaching field. Consequently, apparent alignment between ORF and PRF should not be interpreted uncritically as harmonious or symmetrical. The fact that ORF and PRF seem to promote similar values and contribute to higher compliance with children's rights may mean that one recontextualizing field has become dominant, shaping the terms under which children's rights are understood and implemented. The results of the hierarchical multiple regression suggest that contextual conditions associated with the PRF play a particularly salient role in predicting coaches' compliance, as the effect of formal coach education—an expression of the ORF—loses statistical significance once contextual variables such as type of sport and type of sports club are taken into account. This pattern indicates that the influence of official pedagogic discourse may depend on whether, and how, it is taken up and recontextualized within concrete coaching environments. Although previous research suggests that coach education has traditionally prioritized sport-specific and performance-related knowledge rather than socio-pedagogical content (48), there remains limited empirical knowledge regarding how children's rights are explicitly addressed within coach education and how this influences coaches' compliance with the UNCRC.

The finding that female coaches reported higher compliance across multiple UNCRC dimensions is strongly aligned with established research on gender and coaching styles. Previous studies have shown that women coaches tend to adopt more democratic, inclusive, and athlete-centered approaches, emphasizing communication, care, and relational sensitivity (35, 36). Such approaches resonate closely with key UNCRC principles, including participation (Article 12), protection from harm (Article 19), and the prioritization of children's best interests (Article 3). Importantly, the regression analysis shows that gender remains a significant predictor even when contextual factors are accounted for, suggesting that pedagogic dispositions shaped by gendered socialization retain explanatory power beyond situational characteristics.

From the perspective of Bernstein's pedagogic device (5), this aligns with the idea that policy translation is not neutral but is shaped by individual interpretations. Coaches, as both receivers and agents of policy, filter their understanding of the UNCRC through personal ideologies, which are influenced by broader socialization processes. Gendered socialization may lead male and female coaches to develop different perspectives on the purpose of sports, which in turn affects how they recontextualize and enact the UNCRC in practice. However, rather than attributing these differences solely to gender, it is important to recognize the role of structural and cultural factors, such as coach education, and institutional expectations. Moreover, coaching styles exist on a spectrum, with variations within genders—many male coaches also adopt child-cantered approaches, just as some female coaches emphasize performance outcomes.

### Contextual factors and interpretation of the UNCRC

Beyond individual background factors, the study demonstrates that coaching contexts significantly shape how children's rights are enacted. Coaches working in individual sports reported higher compliance than those in team sports across several UNCRC dimensions, including participation, protection, and health-related practices. This finding aligns with previous research indicating that team sport environments often foster more hierarchical and authoritarian coaching cultures, whereas individual sports may provide greater opportunities for personalized interaction and athlete voice (33, 49). One possible explanation is that individual sports often involve smaller athlete-to-coach ratios, creating more opportunities for personalized interactions. This suggests that the PRF (6) within sports clubs differs depending on the structure and methodology of the sport itself. Coaches working in individual sports may have greater autonomy in implementing policies related to children's rights, whereas team sports, with their hierarchical and collective structures, may reinforce different priorities.

Similarly, coaching younger children and mixed-gender groups may also create contexts in which developmental sensitivity, inclusivity, and flexible pedagogies are more strongly emphasized. These environments may reduce tension between UNCRC principles and everyday coaching demands, facilitating greater alignment between policy intentions and practice. Such findings echo earlier studies showing that children's opportunities for participation and protection vary depending on sport-specific and institutional conditions (11, 12).

The hierarchical regression analysis further reinforces the centrality of context by showing that contextual variables explain additional variance in compliance beyond individual background characteristics, and that type of sport and type of sports club remain significant predictors in the final model. This finding empirically supports the notion that compliance with the UNCRC is not primarily determined by who the coach is, but by where and under what conditions coaching practice takes place.

The finding that coaches from elite clubs reported higher compliance with the UNCRC warrants particular attention. Previous research has frequently identified elite sports clubs as environments where young athletes may be at risk of violations of children's rights [e.g., (20, 50)]. However, the present study's findings indicate that coaches from elite clubs demonstrate greater self-reported compliance with the UNCRC. One possible reason is that elite clubs provide a context in which UNCRC principles are clearly articulated, actively monitored, and integrated into daily practice. Additionally, these clubs typically have access to enhanced resources, such as highly educated staff, which can facilitate more effective understanding and implementation of the UNCRC. Although high-performance settings inherently present distinctive risks [e.g., (20, 50)], this study suggests that robust organizational governance enables elite clubs to foster conditions conducive to the faithful implementation of UNCRC principles within daily coaching activities. Rather than contradicting earlier research, these findings indicate that organizational capacity and policy infrastructure within elite clubs may support the systematic implementation of children's rights, even within performance-oriented environments.

From a Bernsteinian (6) perspective, this pattern highlights the importance of examining power relations between the ORF and the PRF across different sporting contexts. In elite sport environments, where organizational structures are highly formalized and closely aligned with national sport policy, safeguarding frameworks, and regulatory mechanisms, the ORF may exert a stronger directive influence over the PRF. In such contexts, children's rights principles articulated at policy and education levels may be more systematically translated into local coaching practice, contributing to higher reported compliance. By contrast, in recreational or less formalized club environments, the PRF may operate with greater autonomy, allowing locally embedded coaching norms—whether child-centered or performance-oriented—to play a more decisive role in shaping how the UNCRC is interpreted and enacted. Alignment between ORF and PRF should therefore not be understood as uniform synergy, but as a context-dependent outcome shaped by shifting power relations between official policy discourse and localized pedagogic practice.

### Practical significance and implications

The effect sizes of the mean comparisons (e.g., *d* = .39) indicate small differences between groups. Although these effect sizes are modest, they nonetheless represent statistically reliable differences in coaching behavior. Moreover, in fields concerned with children's welfare, development, and rights, even small behavioral differences may have substantial implications when they are repeatedly enacted over time (51). Coaching practices are not isolated events but are embedded in everyday interactions that unfold across training sessions, competitions, and entire seasons. Recurrent differences in how coaches communicate with children, involve them in decision-making, respond to mistakes, or prioritize well-being over performance may gradually accumulate, shaping children's experiences, learning opportunities, and sense of agency within sport. Over time, such cumulative exposures can meaningfully influence children's psychosocial development, perceptions of safety and inclusion, and their relationship to sport more broadly. For example, a small to moderate effect size may reflect consistent differences in how male and female coaches prioritize and enact child-centered practices, which—through sustained interaction—can affect children's well-being, motivation, and developmental outcomes in sporting environments.

Taken together, the findings underscore the need for policy efforts that move beyond formal adoption of children's rights frameworks and attend more closely to the pedagogic processes through which these rights are interpreted and implemented. Strengthening coach education, supporting reflective coaching practice, and addressing contextual constraints within specific sports and club environments appear crucial for advancing the implementation of children's rights in sport. Moreover, by empirically demonstrating how children's rights are localized through coaching practice, this study contributes to emerging context-specific approaches within children's rights research and extends them into the field of sport.

## Limitations and future directions

The study utilized self-reported questionnaires, which come with certain limitations. Such instruments may restrict response options, potentially limiting participants' ability to articulate more nuanced views of their coaching practices. Moreover, responses may be influenced by social desirability bias (52), whereby individuals consciously or unconsciously portray themselves in a more favorable manner. This risk may be particularly salient given that questionnaire distribution occurred through NSOs, which could reinforce normative expectations related to children's rights and appropriate coaching behavior. However, participation was anonymous and voluntary, and no responses were accessible to NSOs or sports clubs. While these measures are likely to mitigate socially desirable responding, the findings should nonetheless be interpreted as reflecting coaches' self-reported understandings and perceptions rather than observed behavior.

In addition, the measure of compliance employed in this study captures the reported frequency of rights-aligned practices, but does not assess the quality, depth, or cultural embeddedness of children's rights implementation within coaching environments. Consequently, higher levels of reported compliance should not be interpreted as evidence of fully realized or deeply institutionalized children's rights cultures in sport. Rather, the findings provide insight into patterns of behavioral–procedural compliance, leaving more interpretive, pedagogic, and ethical dimensions of children's rights practice beyond the scope of the present study.

Another limitation concerns the cross-sectional design, which does not allow for conclusions regarding causal relationships or changes over time. Given that children's rights implementation in sport is likely shaped by cumulative experiences and ongoing pedagogic processes, longitudinal research would be valuable for examining how coaches’ practices develop in relation to education, experience, and changing contextual conditions. Such approaches would enable a deeper understanding of how small differences in coaching behaviors may accumulate over time and influence children's sporting experiences and development.

Future research should therefore complement self-report questionnaires with qualitative and observational methods, such as interviews, ethnographic studies, and practice-based observations, to capture the meaning-making processes through which children's rights are interpreted and enacted in everyday coaching practice. In line with Bernstein's (5) pedagogic device, further studies could more explicitly examine how children's rights are addressed within the ORF (e.g., coach education curricula, policy documents) and how these discourses are taken up, transformed, or resisted within the PRF of sports clubs and coaching environments.

Finally, while the present study provides empirical insights from the Swedish sport context, future comparative research across different national sport systems would be valuable. Given the near-universal ratification of the UNCRC, cross-national studies could deepen understanding of how diverse policy frameworks, governance structures, and coaching cultures shape the localization of children's rights in sport. Such work would further strengthen the international relevance of context-specific approaches to children's rights and support the development of more nuanced and effective strategies for their implementation in coaching practice.

## Conclusion

This study highlights the inherently context-specific nature of children's rights in sports and the persistent challenges of achieving a uniform implementation of the UNCRC. The findings show that coaches' gender, education, and coaching experience, alongside athletes' gender and age and the type of sport and club, all influence how coaches interpret and implement children's rights. However, the regression analysis demonstrated that contextual factors play a particularly salient role in explaining differences in compliance beyond individual background characteristics. In this regard, coaches' gender, type of sport, and type of sports club emerged as the most robust predictors of compliance.

These findings underscore that compliance with the UNCRC cannot be understood as the outcome of formal policy directives alone (Bernstein's ORF), but must be situated within the concrete contexts in which coaching practice unfolds (Bernstein's PRF). The loss of significance of coach education once contextual variables were taken into account suggests that the influence of formal education is not automatic, but mediated through local sport structures, cultures, and organizational conditions. Rather than positioning coach education as a uniform or deterministic driver of children's rights implementation, the findings suggest that it functions as one component of a broader recontextualization process in which policy principles are interpreted and translated into pedagogic orientations. Coaches who reported higher levels of education also reported greater alignment with children's rights principles, indicating that formal education may provide conceptual resources and ethical vocabularies relevant for UNCRC implementation. However, the observed variation across sporting contexts indicates that the impact of coach education is contingent upon how such knowledge is taken up and supported within everyday coaching environments. In this sense, education does not “transfer” policy into practice, but interacts dynamically with coaches' experiences, sport-specific cultures, and local club conditions.

Taken together, the findings reinforce the importance of conceptualizing children's rights in sport not as a fixed standard to be uniformly imposed, but as a pedagogic process shaped by the interplay of policy, people, and place. By empirically demonstrating how UNCRC compliance varies across coaching backgrounds and, more importantly, across sporting contexts, this study contributes to emerging context-specific approaches to children's rights and extends them into the field of sport. These insights are particularly relevant for policymakers, coach educators, and sport organizations seeking to strengthen children's rights implementation, as they highlight the need to address not only formal education and policy frameworks, but also the contextual conditions under which coaching practice is implemented.

## Data Availability

The datasets presented in this article are not readily available because the data are part of a PhD project, and they will be part of future analyses and publications thus, they cannot be shared at the moment. Requests to access the datasets should be directed to georgios.pavlogiannis@umu.se.
